# Fully Automated On-Chip Imaging Flow Cytometry System with Disposable Contamination-Free Plastic Re-Cultivation Chip

**DOI:** 10.3390/ijms12063618

**Published:** 2011-06-07

**Authors:** Masahito Hayashi, Akihiro Hattori, Hyonchol Kim, Hideyuki Terazono, Tomoyuki Kaneko, Kenji Yasuda

**Affiliations:** 1 Kanagawa Academy of Science and Technology, KSP East 310, 3-2-1 Sakado, Takatsu-Ku, Kawasaki 213-0012, Japan; E-Mails: hayashi.bmi@gmail.com (M.H.); hattori.bmi@gmail.com (A.H.); kim.bmi@gmail.com (H.K.); terazono.bmi@gmail.com (H.T.); 2 Institute of Biomaterials and Bioengineering, Tokyo Medical and Dental University, 2-3-10 Kanda-Surugadai, Chiyoda, Tokyo 101-0062, Japan; E-Mail: kaneko.bmi@tmd.ac.jp

**Keywords:** imaging cytometry, on-chip cytometry, cell sorting using microgel electrophoresis

## Abstract

We have developed a novel imaging cytometry system using a poly(methyl methacrylate (PMMA)) based microfluidic chip. The system was contamination-free, because sample suspensions contacted only with a flammable PMMA chip and no other component of the system. The transparency and low-fluorescence of PMMA was suitable for microscopic imaging of cells flowing through microchannels on the chip. Sample particles flowing through microchannels on the chip were discriminated by an image-recognition unit with a high-speed camera in real time at the rate of 200 event/s, e.g., microparticles 2.5 μm and 3.0 μm in diameter were differentiated with an error rate of less than 2%. Desired cells were separated automatically from other cells by electrophoretic or dielectrophoretic force one by one with a separation efficiency of 90%. Cells in suspension with fluorescent dye were separated using the same kind of microfluidic chip. Sample of 5 μL with 1 × 10^6^ particle/mL was processed within 40 min. Separated cells could be cultured on the microfluidic chip without contamination. The whole operation of sample handling was automated using 3D micropipetting system. These results showed that the novel imaging flow cytometry system is practically applicable for biological research and clinical diagnostics.

## 1. Introduction

In biological research and clinical diagnostics, the purification of desired kind of cells from a crude sample mixture is the first and important step for precision analysis. However, the operation of cell purification is time-consuming and contaminating process. Efficient and rapid sorting of cells has been accomplished with techniques such as the fluorescence-activated cell sorter (FACS) [1], the magnetic activated cell separator (MACS) [2], the automated single-cell sorting using dual-beam optical trapping, differential adhesion cell sorting [3], a disposable microfabricated fluorescence-activated cell sorter (μFACS) [4–6]. Recent advancements in optofluidic flow cytometer, where optics and microfluidics work together to create novel functionalities on a small chip, holds great promise for lab-on-a-chip flow cytometry [7,8]. The development of a low-cost, compact, handheld flow cytometer and microfluorescence-activated cell sorter system could have a significant impact in the field of point-of-care diagnostics, improving health care. The traditional flow cytometer and the FACS detect and analyze optical signals (angular light scatter or emitted fluorescence) to identify individual cells or biological samples, and are widely used as analysis tools in biomedical research and clinical diagnostics. These tools greatly facilitate the study of both physical properties (e.g., size, shape) and biochemical properties (e.g., cell cycle distribution, DNA contents) of biological samples such as cells. Information about the cells of interest is obtained optically in a nondestructive and quantitative manner [9,10]. However, FACS can damage cells during destructive droplet generation, and the detection (based on the non-direct scattering) has poor cell recognition performance. Other conventional techniques also have disadvantages with regard to their cost, efficiency, response speed, separate resolution, and adaptability. Furthermore, if cells are to be used for cultivation after purification, the damage on cells caused by the sorting process should be minimized. For example, the MACS method allows cells to be separated by incubating with magnetic nanoparticles coated with antibodies against a particular surface antigen. This causes the cells expressing this antigen to attach to the magnetic nanoparticles. Afterwards the cell solution is transferred on a column placed in a strong magnetic field. In this step, the cells attached to the nanoparticles (expressing the antigen) stay on the column, while other cells (not expressing the antigen) flow through. Although this method is simple and economical, the decorated antibodies can hardly be removed from the cell surface and sometimes these attached antibodies promote the changes of target cells.

In contrast, advantages offered by application of microfluidics are reduced sample volumes, shorter handling time, lowered space requirements and operational costs. Especially, the small size and potentially low cost of μFACS system promote portability and affordability by individual laboratories and point-of-care clinics. There are three core components of μFACS systems that need to be miniaturized while maintaining performance: (1) The fluidic system for introduction and placement of biological samples; (2) The optical system for illumination of samples and collection of emitted light (both scattered and fluorescent); and (3) The sorting system for deflection of samples of interest, which requires both real-time control and a rapid-response actuation system. The key challenge to developing high-performance μFACS system is to figure out how to reduce the volume and cost of these three systems while maintaining adequate performance (e.g., detection sensitivity and sorting throughput). However, even above μFACS system is hard to be applied for non-invasive cell collection of target cells for such like regenerative medicine as far as they use the fluorescent antibodies for labeling and identification of target cells.

We have developed a series of on-chip high speed imaging cell sorting system to be able to overcome problems associated with the conventional cell sorting techniques [11–13]. The system has the following five core features: (1) The fully automated application and collection of samples using 3-D micropipetting system; (2) The whole fluidic system arranged in a disposable plastic chip for lining-up the biological samples in laminar flow using hydrodynamic focusing; (3) The optical system for direct observation-based cell identification with specific image indexes using phase-contrast/fluorescence microscopy, real-time image processing; (4) Non-destructive wider-size dynamic range sorting procedure using mild electrostatic force in a laminar flow exploiting agarose gel electrode to prevent loss of electrode nor any electrolysis bubble formation; (5) Contamination-free recultivation microreservoir for collected target cells. The procedure of mild electrostatic force separation is by applying electrostatic force only to the cells which have to be removed as waste, *i.e.*, the target cells simply flow down the laminar flow and do not receive any stimulation from electrostatic force. In other words, to accomplish the cell sorter system, we need to combine different technologies into a system to be able to acquire particular target cells.

In this paper, we report an automated imaging flow cytometry system using a plastic chip, in which a disposable contamination-free recultivation function was included for simple and safe operation in biological research and clinical diagnostics.

## 2. Results and Discussion

### 2.1. Fully Automated On-Chip Imaging Flow Cytometry System with Disposable Contamination-Free Plastic Re-Cultivation Chip

**Figure 1:**
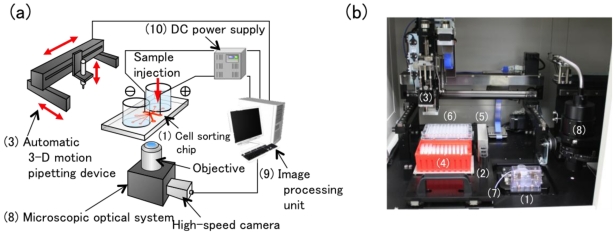
Fully automated on-chip imaging flow cytometry system with disposable contamination-free plastic re-cultivation chip. (**a**) Schematic diagram of the system; (**b**) Layout of components in the system.

We have developed a fully automated system of on-chip imaging flow cytometry system containing: (1) A cell sorting chip attached to a cell-sorter chip holder; (2) A holder of sample tubes and collection tubes; (3) An automatic 3-D pipetting device; (4) A holder of pipette chips; (5) An ejection port for used pipette chips; (6) Air pressure unit connected to a syringe pump; (7) An automatic alignment set of phase-contrast/fluorescent optical microscopy module; (8) An image-processing unit consisting of a high-speed camera and an image-processing and system control computer; (9) A switching DC power supply (Figure 1a, b).

**Figure 2:**
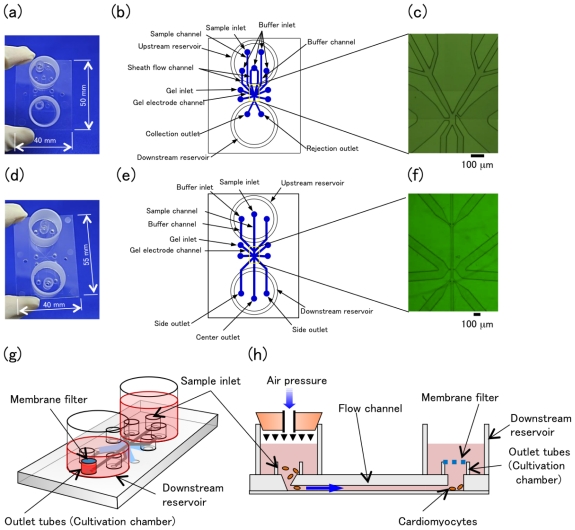
Two- (**a**–**c**) or three- (**d**–**f**); way-outlet type cell-sorter chips. Full view (**a** and **d**), schematic diagram (**b** and **e**), and close-up images (**c** and **f**) of the chips. (**g**) Full view and (**h**) side view of a re-cultivation chip.

As shown in Figure 2, all the microfluidic pathways for cell sorting were arranged in the cell sorting chip.

### 2.2. Design of Cell-Sorter Chips

We have fabricated two types of cell-sorter chips; a two-way-outlet type cell-sorter chip (Figure 2a–c) and a three-way-outlet type cell-sorter chip (Figure 2d–f). Both cell sorter chips have the following parts: the inlet pathways of samples and buffers, hydrodynamic focusing pathways, cell sorting area where sample and buffer flows join together while keeping their boundaries, and gel channels (a pair of agarose microelectrode storage areas) attached to the cell sorting area, and the outlet pathways of target samples and wastes. The section dimension of the cell-sorting area was 50 μm wide by 25 μm high. At the agarose microelectrode storage area, these two wide spaces were filled by the 1% agarose containing 0.5 M NaCl as electrolyte. The sol–state agarose was injected into the gel inlet on the chip just before each experiment started. Small acrylic tubes were attached on the cell-sorter chip as sample and collection inlet, and large acrylic tubes were also attached to the inlet area at upstream, and outlet area at downstream, for buffer reservoir (Figure 2g–f). In the two-way on-chip cell sorter chip, the raw sample was introduced from a sample reservoir into one of the two microfluidic pathways fabricated in the chip, which shifted the cells to the other buffer pathway when the voltage was applied to gel electrodes. On the other hand, in the three-way on-chip cell sorter, the raw sample was introduced into the center pathway, and shifted to the left or right side laminar buffer flow depending on the charges. It should be noted that the thickness of the bottom, thin PMMA film was 100 μm (which is within a depth of focus of ×100 obj. lens) and was attached to the microfluidic chip by heat bonding.

### 2.3. Automated Sequential Process of Imaging Cytometry

**Figure 3:**
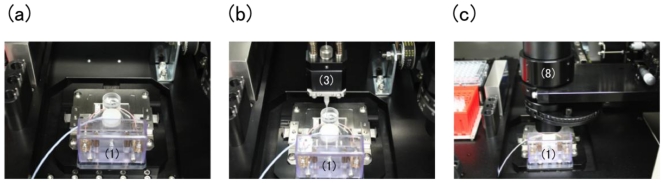
Automatic setup process of the cytometry system: (**a**) Setting of a PMMA chip on a chip; the initial state of the setup process; (**b**) loading of sample and sheath buffer on a sample inlet and a reservoir pipe by automatic pipetting device; and then (**c**) moving of a condenser lens at a measuring position over the chip; the end state of the process.

Before the automatic operation of cell sorting starts, a cell-sorter chip, sample tubes, a sheath buffer tube, collection tubes, and pipette chips were placed on a cell-sorter system by hand (Figure 3a). Then the system cover was closed and the air inside was filtrated and cleaned by HEPA filter-based air circulation. The initialization process was carried out by the system control unit to reset and memorize all the positions and conditions. Then an automatic 3-D pipetting device applied the sample into the sample inlet and the sheath buffer into upstream/downstream buffer reservoirs (Figure 3b), and then an automatic alignment system of optical microscopy system adjusted the positions and focus of objective and condenser lens (Figure 3c). Following the above initialization process, the pressure was applied to the upstream reservoir to start the sample flow and cell sorting process. 1/200 s real time images of sample particles flowing through the cell sorting area were acquired using a high-speed camera and the image-processing computer recognized characteristics of each particle according to the parameters of the particle size and shape, and decided to apply DC voltages into the gel electrodes at the rate of up to 200 particle/s real time. All the images of particles and their parameters were stored in the data storage of the computer.

### 2.4. Real-Time Recognition of the Image of Flowing Particles

**Figure 4:**
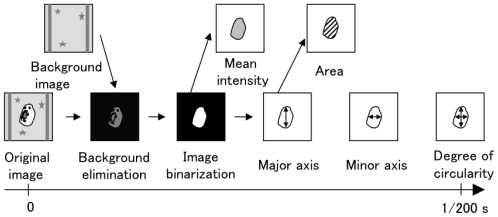
Analysis procedure of microscopic images in real time by image processing unit.

The image processing unit recognized the image of each flowing particle at a rate of 200 frames/s, as shown in Figure 4. A background image of observation area was acquired at the end of initialization process. In the sorting process, to decide the boundary of particle image, an original image was subtracted by the background image in each frame and binarized by the preset threshold. The parameters of the particle’s image, such as mean intensity, area, length of the major and minor axis, and the degree of circularity, were calculated according to the pixel value within the boundary. When one of, or a set of, the parameters satisfied that of a desired or undesired particle, the image-processing unit switched the DC voltage between the gel electrodes of the cell-sorter chip by switching DC power supply in real time.

### 2.5. Development of Adisposable Poly(methyl methacrylate (PMMA)) Imaging Cell Sorter Chip

**Figure 5:**
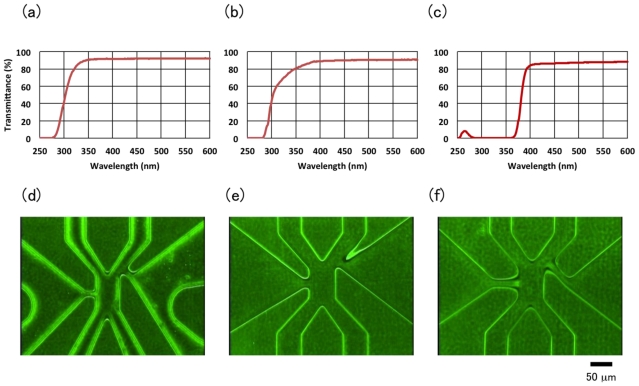
Development of a PMMA-based flammable cell sorter chip. (**a**–**c**) Transmittance of a glass slide (**a**), a polystyrene-based culture dish (**b**), and a PMMA-based cell-sorter chip (**c**), the thickness of all sample plate was 1 mm. (**d**–**f**) Molding of PMMA-based cell sorter chips by hot embossing method (**d**) and injection molding method (**e**, **f**). The mold design of (**f**) was inserted in narrow channel structures between the flow channel and gel channels to the design of (**d**) and (**e**).

Due to advantages such as transparency for visible light, low self-flouorescence, flammability after use, ability of glue-free heat bonding, flexible change of shape by heat if necessary, and hydrophilicity in microfluidic pathway, we chose poly(methyl methacrylate (PMMA)) as the base material of the disposable and economical cell sorter chip. As shown in Figure 5a–c, PMMA has a high transmittance, about 90% at the range of visible light from 400 to 600 nm; the transmittance was approximately the same as a glass slide and a polycarbonate culture dish.

To mold a PMMA-based chip, we first examined the hot embossing method. As shown in Figure 5(d), the molded microchannels showed rounded edges and did not reflect the microscopic range edges of microfluidic pathways. This was because PMMA was not soft enough to permeate the edge of a square-shaped microchannel at the temperature required for the hot embossing process. Next, we examined an injection molding method using the same design of microchannels for the hot embossing process. The edge of the microchannel comprised a sharp square shape, but melting PMMA resin did not permeate the thin wall structure at the junction of the flow channel and gel channel (Figure 5e). Next, we redesigned the injection mold to thicken the wall structure by the addition of narrow channels with 15 μm width and 45 μm length between the flow channels and the gel channels. We succeeded the fine molding of a PMMA-based cell sorter chip with the new design; the PMMA resin permeated into the walls well around all microchannels (Figure 5f). The sectional dimension of the flow channel in the cell sorting area was 50 μm wide by 25 μm high. The molded PMMA plate was laminated with a thin PMMA sheet (100 μm thick) by hot lamination.

### 2.6. Spontaneous Filling of Agarose Sol into the Gel Electrode Channel of PMMA-Based Cell-Sorter Chip

**Figure 6:**
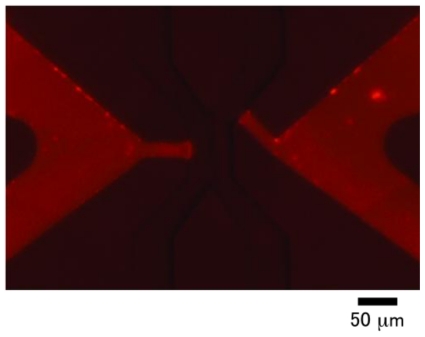
Fluorescent image of the cell sorting area of an injection molded PMMA-based chip which was filled with agarose gel mixed with rhodamine B.

A PMMA-based chip was preheated on a hot plate at 42 °C for 5 min. Melting sol containing 1.5% agarose and 0.5 M NaCl was loaded on the gel inlets on the preheated chip. Unlike the PDMS-based microfluidics chip as described in a previous report [12,13], melting gel spontaneously permeated into the gel channels by capillary phenomena without additional pressure. The injecting sol turned the junction of flow channels and gel channels around, permeated the narrow channel structure, and stopped at the tip of the narrow channel. The agarose sol was turned to gel by cooling at room temperature, keeping the structure attached to flow channels (Figure 6). The spontaneous permeation of melting gel was due to the wettability of PMMA (more than PDMS).

### 2.7. Laminar Flow of Sample Solution Sheathed by Hydrodynamic Focusing of Buffer Streams in the Microchannel of PMMA-Based Cell-Sorter Chip

**Figure 7:**
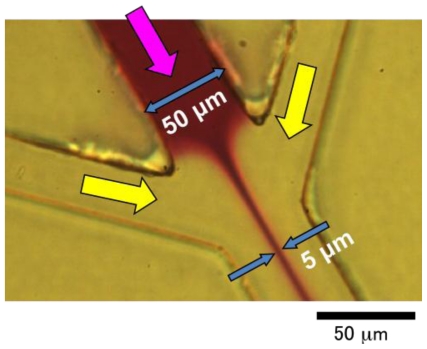
Laminar flow generated by three-forked sheath structure. Sheath buffer containing red ink was applied as sample solution. Flow speed was 1 mm/s.

To examine the laminar flow generation in the microchannel of PMMA-based cell-sorter chip, we loaded a solution containing dark ink at the sample inlet and clear buffer into the buffer inlets (Figure 7). The stream of sample solution was narrowed from 50 μm to 5 μm by hydrodynamic focusing mechanism as described in previous reports [11–13]. The laminar flow of sample and sheath solution was observed without turbulence, nor intermixture, at the flow rate of the cell-sorting area from 0.1 to 200 mm/s (data not shown). The flow rate of the cell-sorting area was about ten times higher than the sample inlet channel, and the linear density of sample particles in the cell-sorting area was ten times lower than the sample inlet channel. This was because the ratio between the width of the laminar flow from the sample channel and sheath channels was 1:10, as described in [13]. The PMMA-based cell-sorter chip had sufficient microchannels to perform on-chip sorting as precisely as the PDMS-based chip.

### 2.8. Particle Sorting and Separation Using Two- and Three-Way-Outlet Type Cell Sorter Chip

**Figure 8:**
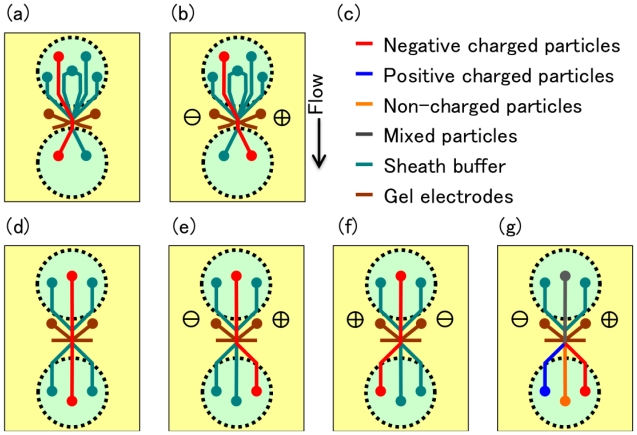

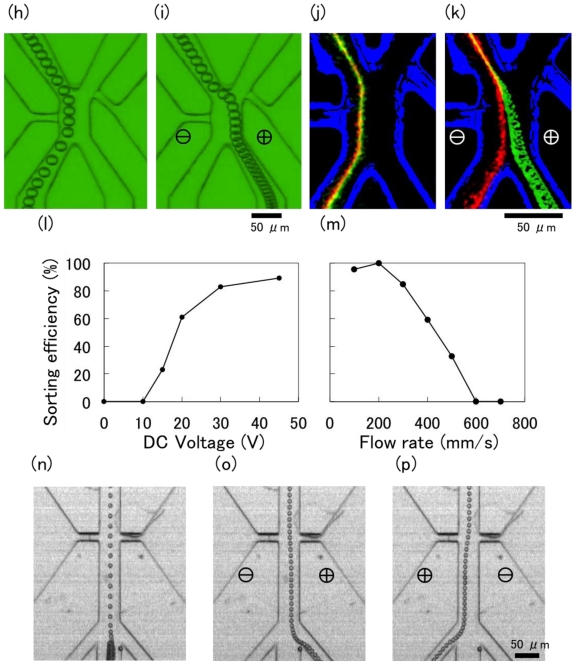
Particle separation using two- or three-way outlet type cell-sorter chip. (**a**–**g**) Switching mechanism of the two types of cell-sorter chips using electrophoresis by gel electrodes. (**a** and **b**) Two-way type and (**d**–**g**) three-way type chip. (**c**) Color codes of the charged state of sample particles and buffers. Sample particles flow along a red line without DC voltage as shown in (**a**) and (**d**). Application of DC voltage switches the outlet channels according to the sign of particle’s charge and the polarity of DC voltage (**b**,**e**, and **f**). Mixed particles of negative, positive, and non-charged particles sorted exclusively to three outlets by DC voltage (**g**). (**h** and **i**) Tracks of a 20 μm polystyrene particle on a two-way outlet type chip applied without (**h**) and with (**i**) DC voltage between gel electrodes. (**j** and **k**) Separation of 2 μm polystyrene particles (green track) and fluorescein (red track) applied without (**j**) and with (**k**) DC voltage between gel electrodes. The dependence of sorting efficiency on the DC voltage between gel electrodes (**l**) and the flow rate (**m**). (**n**, **o**, and **p**) Tracks of a 2 μm polystyrene particle on a three-way outlet type chip applied without (**n**) and with DC voltage in opposite directions (**o** and **p**) between gel electrodes. The direction of electric field was indicated by plus and minus icons on each figure. Flow direction in all figures was from top to bottom.

We manufactured two kinds of PMMA-based cell sorter chips with two- and three-way outlet channels (Figure 8). Sample particles aligned on the centerline of an upstream microchannel using the sheath flow mechanism, and flowed through the exact position of a cell-sorting area between gel electrodes. The outlet channel can be changed using electrophoresis by switching the DC voltage applied on the electrodes while particles are passing between the tips of gel electrodes. In the case of the two-way-outlet type chip, inlet channels had a symmetrical structure (Figure 2a–c, 8a, and 8b). Sample inlet flow was sheathed with two sheath channels. The sheathed channels and their mirror image channels joined at the gel electrode connection point and branched into two symmetrical outlet channels. While the stream of sample particles flowed to the same side outlet when the DC voltage was turned off (Figure 8a), it flowed to the other outlet when the DC voltage was applied between the gel electrodes (Figure 8b). The separation efficiency using a two-way-outlet type cell-sorter chip in the application of continuous DC voltage depended on the voltage and flow rate (Figure 8l, m). At a constant flow rate, the separation frequency increased with the increasing DC voltage and reached a plateau; the efficiency was 90% at a voltage of 45 V and flow rate of 200 mm/s (Figure 8l). On the other hand, at a constant voltage, the separation frequency decreased with an increasing flow rate and no particles were separated; the efficiency was less than 50% at a voltage of 45 V and flow rate of 500 mm/s (Figure 8m). Moreover, the two-way-outlet type chip separated small molecules and charged particles, e.g., the sample mixture of polystyrene particles and fluorescein was exclusively separated (Figure 8j, k).

In the case of the three-forked type chip, two sets of the sheathed triplet channels were symmetrically placed as inlet and outlet channels on the chip (Figure 2d–f, 8d–g). While the stream of sample particles flowed to the center outlet when the DC voltage was turned off (Figure 8d, n), it flowed into one of the other outlets according to the particle charge and the direction of the electric field in the application of the DC voltage between the gel electrodes (Figure 8e–f, 8o–p). Mixed suspension that contained negative, positive, and non-charged particles was separated into three outlet channels according to the charged state of each particle (Figure 8g). This is an advantage of the three-way type chip for the purification of desired cells from crude samples containing various kinds of undesired particles such as cells, debris, and inanimate particles.

### 2.9. Real-Time Size-Discrimination of Two Similar Sized Particles by Microscopic Image

**Figure 9:**
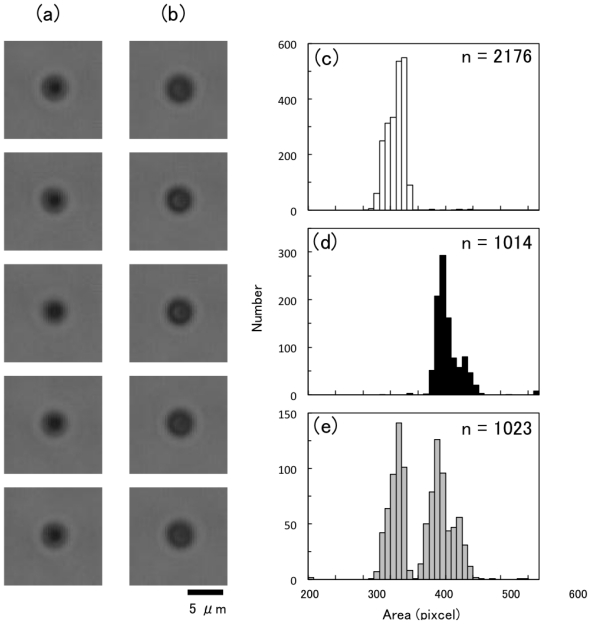
Real-time size-discrimination of two similar sized particles by microscopic image. (**a** and **b**) Bright-field microscopic images of polystyrene particles with 2.5 μm (**a**) and 3.0 μm (**b**) in diameter. (**c**–**e**) Flow rate was 10 mm/s. Histograms of particles’ area measured by the imaging cytometry system. Sample suspension containing only 2.5 μm particles (**c**), only 3.0 μm particles (**d**), and mixed suspension with 2.5 and 3.0 μm particles (**e**).

To examine the image-recognition ability of the cytometry system, we applied the mixed suspension containing two kinds of similar sized particles (Figure 9). The images of particles were clear but hard to discriminate by human eye without scale or size-comparison subjects (Figure 9a, b). However, the image recognition unit of the cytometry system discriminated precisely polystyrene particles with 2.5 μm or 3.0 μm in diameter with a less than 2% error at a rate of 200 frames/s in real time, at a flow rate of 1 mm/s (Figure 9c–e). In the case of the sample mixture with 5 μm and 10 μm polystyrene, the discrimination error was less than 1% at a flow rate of 200 mm/s (data not shown). For the mixture containing two kind of particles with given diameters, the discrimination error increased with increasing flow rate as described in previous work [13]. These results suggests that imaging cytometry displays a lot of advantages for the precise and high-throughput separation of desired cells from undesired cells with much the same size indistinguishable by the human eye.

### 2.10. Estimation of the Maximum Processing Speed and the Necessary Time for Cell Sorting

Estimating the performance of the novel cytometry system based was then possible with the following specifications as described above: Maximum sampling rate of image acquisition and recognition was 200 events/s. The sorting efficiency was 90% at a flow rate of less than 200 mm/s in the cell-sorting area. The dimensions of the cell-sorting area were 50 μm in width, 25 μm in height, and 100 μm in length. The flow rate at the cell-sorting area was 10 times higher than in the sample inlet because of a sheath structure. The discrimination error was less than 2% for the mixture of 2.5 μm and 3.0 μm polystyrene particles. The whole operation after sample setting was automated and took less than 5 min excluding the cell sorting process.

Since the maximum sampling rate was 200 events/s, the maximum sample-processing rate was 2.5 nL/s (= 50 μm × 25 μm × 100 μm × 200 event/s ÷ 10). Under these conditions, the flow rate at the cell-sorting area was 20 mm/s (= 100 μm × 200 events/s), which is lower than the maximum flow rate for precise cell sorting, 200 mm/s. For example, 5 μL of sample will be processed within 33 min (= 5 μL ÷ 2.5 nL/s). Then the total operation time is less than 40 min.

Because the unit volume of sample processing was very small (125 pL/event, the volume of the cell-sorting area per event), the sample dilution was not necessary for cytometry; Maximum density of sample particle is 8 × 10^6^ particles/mL (= 1 ÷ 125 pL). For example, if the sample density is 1 × 10^6^ particles/mL, that is 1/8 of the maximum density, the sample particles are processed one by one with the entanglement error in cell sorting less than 0.8 % according to Poisson’s distribution.

Therefore, to analyze a sample of 5 μL with 1 × 10^6^ particle/s, take the desired cells with discrimination error less than 2% in a collection tube and the database of microscopic image checks all the cells within 40 min automatically. These results demonstrate that the novel cell-sorting system outlined in this paper is useful for use in biological research and clinical diagnostics.

### 2.11. Sealing of Semipermeable Membrane on the Chip Reservoir for Contamination-Free Recultivation Chip

Transfer of sorted cells from the cell-sorter chip into another culture vessel involves risk of contamination, damage, and loss of cells. To overcome these risks, we developed the on-chip culture system using a PMMA-based cell-sorter chip. Although the sorted cells in the cell-sorter chip can be cultured by the transfer of the whole chip into an incubator, there was the risk of contamination because the medium in outlet tubes are exposed to outside air. Moreover, since the two or three outlet tubes on a cell-sorter chip were linked by culture medium, the waste cells or particles can contaminate the collection outlet by convection or self-propulsion during cultivation. To prevent contamination mediated by culture medium, we tried to separate outlet tubes and a downstream reservoir by a membrane filter. The membrane filter needs the permeability of nutrients and gasses and the impermeability of cells and bacteria. We tried to tightly seal between the edge of an outlet tube and a membrane by thermal bonding. Since the softening point of PMMA is about 100 °C, a membrane filter should maintain resistance at the necessary temperature for thermal bonding. For tight sealing, the membrane must not have a ruck on its surface. The permeability of nutrients and gasses require the wettability of a membrane for culture medium. The clarity for visible light allows us to observe the cells in outlet tubes without breaking the seal. We compared seven commercial membrane filters using the criteria described above, and selected four candidates (Table 1).

**Figure 10:**
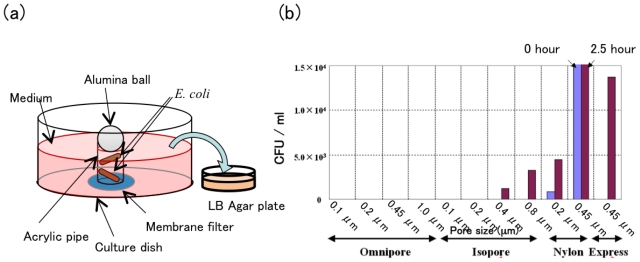
Permeability of *E. coli* through membrane filters bonded on a dummy outlet tube by thermal bonding: (**a**) Experimental setup; (**b**) Number of colonies on LB agar plated in the outside medium, after the injection of *E. coli* suspension into a dummy outlet tube.

Next, the permeability of *E. coli* through the membrane filter to bond on the edge of outlet tubes was estimated using dummy tubes. Although permeability of *E. coli* was not observed using the Omnipore filter for all pore sizes and Isopore filter with pore sizes of 0.1 and 0.2 μm, Isopore with pore sizes more than 0.4 μm and the other types of filters had permeability (Figure 10).

**Figure 11:**
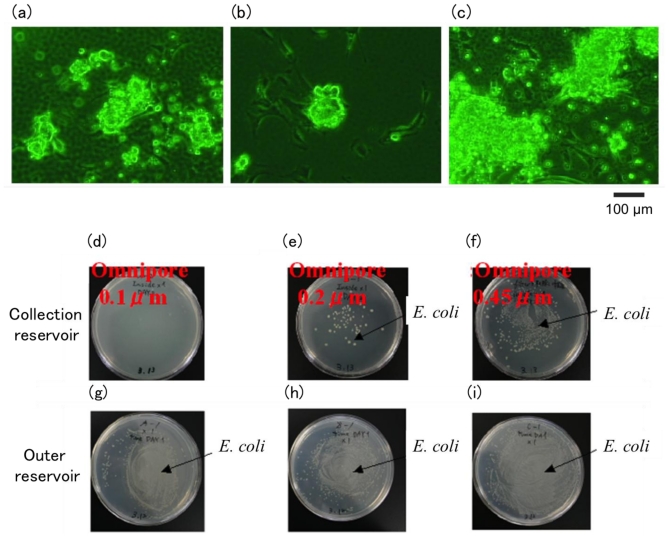
Estimation of viability of cells and contamination from downstream reservoir using a PMMA-based cell-sorter chip with separation filters. (**a**–**c**) Cardiomyocyte cultured in the bottom of an outlet tube after cultivation for two days. Most cells were beating. LB agar plated in the medium, in outlet tubes (**d**–**f**) and downstream reservoir (**g**–**i**) after cultivation for two days. The cultivation chip was sealed on its outlet tubes by Omnipore filters: with a pore size of 0.1 μm (**a**, **d**, and **g**); 0.2 μm (**b**, **e**, and **h**); or 0.45 μm (**c**, **f** and **i**).

Further, we investigated the properties of a cell-sorter chip whose outlet tubes were sealed using an Omnipore filter (Figure 11). Cardiomyocyte showed the adhesion to the bottom of membrane-sealed outlets and beating over two days. The contamination of *E. coli* from downstream reservoir into outlet tubes during cultivation was observed using Omnipore filters with a pore size of 0.2 μm and 0.45 μm, but was not observed with 0.1 μm pores. Therefore the PMMA-based cell-sorter chip with separation filter on the edge of outlet tubes is useful for on-chip cultivation without contamination.

## 3. Experimental Section

### 3.1. System Configuration of an Automatic On-Chip Imaging Cytometer

The configuration of a fully automated system of on-chip imaging cytometry was described in Section 2.1 as shown in Figure 1. The optical system constituted a halogen lamp (LG-PS2, Olympus, Japan), a condenser lens (IX-LWUCD, Olympus, Japan), an objective lens (LCPlanFl 20× or LUCPlanFlN 40×, Olympus, Japan), a camera lens (U-TLU, Olympus, Japan). Only for the real-time imaging cytometry (data shown in Figure 4), 40× objective was used. The high-speed camera acquired images at 200 frames/s (HAS-200, Ditect, Japan).

### 3.2. Estimation of a Contamination-Free Re-Cultivation System on a Cell-Sorter Chip

We compared the following commercial membrane filters as a separator between the outlet and reservoir of a cell-sorter chip: Durapore, Isopore, Omnipore, Express, Fluoropore, PVC, Nylon. Membrane filters were sealed on the edge of an outlet tube or a acrylic dummy tube with the same size of outlet tube (6 mm outside diameter, 4 mm inside diameter, 5 mm in length) by thermal bonding using a hot iron for 5 s at 110 °C, which is a temperature just above the softening point of PMMA. For all the experiments in this subsection, no antibiotic was added to culture medium and plates. To examine the tightness of sealing between an outlet tube and a membrane filter, a membrane-sealed dummy tube was injected with *E. coli* suspension in LB buffer, soaking the membrane side into the fresh LB medium, and incubated at room temperature for the given time. The outside medium was plated on 1.5% LB agar plate and cultured at 37 °C for two days. Number of visible colonies on the plate was counted as the index of cell permeability of the membrane. The cell-sorting chip, with membrane sealing on outlet tubes, was constructed as follows: after filling gel electrodes and gluing reservoir tubes on a cell-sorting chip, membrane filters were sealed at outlet tubes by thermal bonding as described above. Culture medium without antibiotic was loaded into the upstream reservoir and injected by air pressure until the outlet tubes were filled, and medium was loaded into the downstream reservoir. To exam the on-chip cultivation in the membrane-sealed outlet tube without contamination, mouse primary cardiomyocytes were applied into an inlet tube of the chip which flowed into the outlet tubes due to air pressure. After the addition of *E. coli* suspension into the downstream reservoir, the cell-sorter chip was incubated under the outlined conditions with 5% CO_2_ at 37 °C for two days. The condition of the cultured cardiomyocyte was checked by the adhesion onto the chip surface and the beating using a phase contrast microscope. To check the contamination of *E. coli* into the outlet tubes, after washing the downstream reservoir with 3 mL of fresh medium ten times, the outside medium in the reservoir was removed, the membrane was pricked with a tip of micropipette, inside medium in the outlet tube was picked up by the micropipette, and the contamination number of *E. coli* in the inside medium was estimated by plating on LB agar plate as described previously.

### 3.3. Samples

Polystyrene particles were purchased from Polysciences, USA. Sample suspending buffer and sheath buffer was double distilled water. Glass slide (S-1111, Matsunami, Japan) and polystyrene-based culture dish (1820-024, Iwaki, Japan) were used for the comparison of transmittance with PMMS-based cell-sorter chip. All of the sample materials had a thickness of 1 mm. Absorption spectra were acquired by U-3010 spectrometer (Hitachi, Japan). For visualization of gel electrode or sheathed sample stream, about 1% rhodamine B (R-6620, Sigma, USA) was added in agarose or sheath buffer. Fluorescence images were acquired by IX70 microscope (Olympus, Japan).

## 4. Conclusions

We developed a novel imaging cytometry system using a PMMA-microfluidic chip. The advantages of this system are: (1) Optical image-based real time cell sorting; (2) All cell sorting procedures included in an economical and disposable (flammable after use) plastic chip; (3) Cell separation electrodes consist of stable and bubble formation-resistant gel-electrodes; (4) Contamination-free re-cultivation reservoir; and (5) Fully-automated operation. The results also indicated that cells and fluorescent dyes can be separated using this system. The necessary time for cell sorting from a sample with 1 × 10^7^ particles and 20 μL is within 30 min. Therefore this simple, safe, fast and economical flow cytometry system with the above advantages is practical and useful for use in biological research and clinical dignostics.

## Figures and Tables

**Table 1:** Comparison of seven types of commercial membrane filters as separation filter for on-chip cultivation.

	Omnipore	Express	Nylon	Durapore	Isopore	Fluoro-pore	PVC
Thermal resistance	×	○	○	○	○	×	×
Ruck	○	○	○	○	×	×	×
Hydrophilia	○	○	○	×	×	×	×
Clarity	○	×	×	×	×	×	×
Result	○	×	×	×	×	×	×
